# The associations between nailfold microvascular abnormalities and systemic inflammation or Th17/Treg dysregulation in rheumatoid arthritis

**DOI:** 10.3389/fimmu.2026.1848275

**Published:** 2026-06-26

**Authors:** Zheng Wang, Yi Tian, Jinglei Wang, Yida Xing, Xiaodan Kong

**Affiliations:** Department of Rheumatology and Immunology, the Second Hospital of Dalian Medical University, Dalian, China

**Keywords:** immunity, inflammation, nailfold microcirculation, rheumatoid arthritis, Th17/Treg ratio

## Abstract

**Objectives:**

To investigate the abnormal characteristics of nailfold microcirculation (NMC) in patients with rheumatoid arthritis (RA) and to further analyze the associations between these abnormalities and common systemic inflammatory indicators or the key immunoregulatory cell subpopulation Th17/Treg cells.

**Methods:**

This cross-sectional observation study enrolled 120 RA patients as the RA group and 60 gender- and age-matched healthy individuals as the healthy control (HC) group. A NMC microscope was used to assess the NMC status and clinical data were collected. Spearman correlation was used to evaluate the relationships between NMC abnormalities and inflammatory or immunological indicators. Lasso regression and random forest model were used to screen key predictors. Receiver operating characteristic (ROC) curves were used to assess the diagnostic efficacy of the Th17/Treg ratio for moderate-to-severe NMC abnormalities, and the goodness-of-fit and clinical utility were validated using the Hosmer-Lemeshow test, calibration curves, and decision curve analysis (DCA). Bootstrap method was used for internal validation.

**Results:**

Compared to the HC group, the RA group possessed higher number of crossed capillary loops, morphology score, flow pattern score, perivascular score, and total NMC score but lower flow velocity. Spearman correlation analysis demonstrated that the total NMC score was positively correlated with the platelet-to-lymphocyte ratio (PLR) (*p* = 0.028), platelet-to-red blood cell ratio (PAR) (*p* = 0.037), systemic immune-inflammation index (SII) (*p* = 0.009), and interleukin-2R (IL-2R) (*p* = 0.046). A total NMC score >4 was defined as moderate-to-severe NMC abnormality. Lasso regression and random forest model identified Th17/Treg ratio was the only independent associated factor for this subgroup and the area under the curve (AUC) was 0.708. The Hosmer-Lemeshow test and calibration curve confirmed favorable goodness-of-fit, and the DCA curve confirmed considerable clinical benefit of this model. The mean AUC from internal validation was 0.706, indicating satisfactory model stability.

**Conclusions:**

Our study illustrated the severity of NMC abnormality was significantly associated with systemic inflammatory burdens (PLR, PAR, SII) and core indicators of T-cell activation and dysfunction (IL-2R and Th17/Treg ratio).

## Introduction

RA is a chronic, systemic autoimmune disease primarily characterized by erosive arthritis. Its pathological basis is synovitis followed by cartilage and bone destruction. Currently, the development of assessment methods for synovitis and bone lesions are rapid. A recent study utilized photoacoustic imaging to measure synovial oxygenation status in RA patients to comprehensively evaluate the correlations between synovial thickening or hypoxia and reduced vascular structures or increased disease activity (1). Another study used high-resolution peripheral quantitative computed tomography to observe sclerotin and microstructure of the bone (2). Since RA is a systemic disease, in addition to local joint lesions, the involvement of vascular system is a common extra-articular manifestation which is one of the most important causes for high disability and mortality rates of RA patients (3). Abundant epidemiological evidence indicated that RA patients had a significantly higher risk of developing cardiovascular disease (CVD) than the general population, and CVD had become a major cause of premature death in RA patients (4).The increased CVD risk associated with RA cannot be fully explained by traditional risk factors (such as hypertension and hyperlipidemia); its roots are deeply embedded in the disease’s intrinsic chronic, systemic inflammation and autoimmune imbalance.

A persistent inflammatory state is closely associated with the activation and injury of vascular endothelial cells, which may contribute to endothelial dysfunction. This process is the initial step in the development of atherosclerosis and may serve as a potential pathophysiological link between local joint inflammation with systemic vascular disease. Under this background, early and non-invasive assessment of the vascular status of RA patients is of paramount importance. As the terminal part of the circulatory system, the microcirculation is a key site for substance exchange and organ perfusion; changes in its structure and function are often the earliest manifestations of systemic vascular disease, potentially even preceding clinically detectable macrovascular lesions (5).Therefore, the microcirculatory system is regarded as an ideal window through which the health status of the entire vascular system can be glimpsed in real-time and non-invasively.

Nailfold videocapillaroscopy (NVC) is a mature, reliable, and reproducible non-invasive imaging technique that allows for direct, *in vivo*, non-invasive observation of the morphology, arrangement, and blood flow state of capillary loops in the dermis papillary layer of the nailfold skin (6). In recent years, researches on the application of NVC in RA had been increasing rapidly. NMC changes in RA patients are typically classified as a non-specific pattern, with main features including twisted loops, morphological diversity, tortuosity and dilation, neovascularization, and perivascular exudate and hemorrhage (7). Despite being labeled “non-specific,” these changes are not without clinical significance at all. They are direct morphological evidences of endothelial injury, inflammatory response, and abnormal tissue repair, reflecting the disease’s underlying vascular pathological activities (8).

Although we know that NMC abnormalities exist in RA patients, the quantitative relationship between the severity of these abnormalities and underlying immunological and inflammatory states driving disease progression still needs further elucidation. The core of this study lies in whether a pathophysiological link can be established between upstream immune imbalance and downstream visible microvascular damage through a series of novel, easily accessible biomarkers. To verify our hypothesis, we utilized systemic immune-inflammatory indicators derived from routine complete blood counts, including the PLR, PAR, and SII (9). These indicators integrate information from neutrophils, lymphocytes, and platelets which together play key roles in inflammation, immune response, and thrombosis of RA patients and are considered as innovative, economical, and convenient tools for assessing the body’s systemic inflammatory burden and immunological status (10).

Among these indicators, SII was thought to have the ability to fully reflect the complex balance between immunity and inflammation (11). Secondly, IL-2R is a direct product and a reliable marker of T lymphocyte activation. Given the central role of T-cell activation in the pathogenesis of RA, serum IL-2R levels can directly reflect the intensity of RA’s core pathological process and have been confirmed to be closely related to disease activity (12). Finally, we focused on the forefront of current immunological researches of RA: the Th17/Treg cell axis. Th17 cells are a potent pro-inflammatory cell subpopulation, whereas Treg cells play a key role in suppressing immune responses and maintaining self-tolerance. One of the core pathological features of RA is a severe shift from the Th17/Treg balance to the pro-inflammatory Th17 subpopulation, which is associated with chronic inflammation, synovial angiogenesis, and osteoarticular tissue destruction (13).

## Materials and methods

### Study population

This study was a single-center, cross-sectional observation study. The study subjects were patients who visited the outpatient or inpatient departments of Rheumatology and Immunology at the Second Hospital of Dalian Medical University between January 2024 and December 2024. The inclusion criteria for the RA group (n=120) were based on the revised RA classification criteria created by American College of Rheumatology (ACR) in 1987 or the RA classification criteria created by ACR/European Alliance of Associations for Rheumatology (EULAR) in 2010 (14). Members of the HC group (n=60) were healthy volunteers undergoing routine physical examinations at the physical examination center of our hospital during the same period, who were screened to ensure no statistical differences in gender composition and age distribution compared to the RA group.

Any individual meeting any of the following criteria was excluded: (a) concurrent diagnosis of other autoimmune diseases, such as systemic lupus erythematosus, systemic sclerosis, Sjögren’s syndrome, etc.; (b) presence of an acute infectious disease within the last 4 weeks; (c) history of malignant tumors; (d) suffering from primary hematological diseases, such as leukemia, essential thrombocythemia, etc.; (e) presence of severe cardiac, hepatic, or renal dysfunctions; (f) suffering from diabetes or ineffectively controlled hypertension; (g) a definite history of smoking (including current smokers and those who had quit for less than one year); (h) usage of drugs that could affect microcirculatory function within 4 weeks prior to the NVC examination, such as vasodilators, calcium channel blockers, etc. (i) presence of Raynaud’s phenomenon (primary or secondary) confirmed by medical history and physical examination. The enrolled RA patients were further divided into two subgroups according to disease course and treatment history: Newly diagnosed RA: disease course <1 year, no prior treatment with DMARDs, glucocorticoids, or biological agents; Long-standing RA: disease course ≥1 year, received standardized antirheumatic treatment. Among the 120 RA patients, 38 (31.7%) were newly diagnosed and 82 (68.3%) were long-standing RA patients. The treatment status of RA patients was classified into four categories according to the 2024 EULAR rheumatoid arthritis treatment guidelines: (a) Untreated (n=38, 31.7%): No prior treatment with any antirheumatic drugs, including DMARDs, glucocorticoids, or biological agents; (b) csDMARDs monotherapy (n=42, 35.0%): Treatment with a single conventional synthetic DMARD, including methotrexate (n=28, 66.7%), leflunomide (n=10, 23.8%), and sulfasalazine (n=4, 9.5%); (c) csDMARDs + low-dose glucocorticoids (n=26, 21.7%): csDMARDs combined with oral prednisone at a dose ≤10 mg/day (equivalent dose); (d) b/tsDMARDs-based therapy (n=14, 11.7%): Treatment with biological or targeted synthetic DMARDs, with or without csDMARDs, including TNF inhibitors (n=8, 57.1%), IL-6 inhibitors (n=4, 28.6%), and JAK inhibitors (n=2, 14.3%). The detailed distribution of treatment status is summarized in **Supplementary Table 1**.

### Collections of clinical data

Nailfold microcirculation was assessed using a TR8000D digital nailfold microcirculation detector (Jiangsu Tongren Medical Electronic Technology Co., Ltd., Xuzhou, China) equipped with a built-in high-resolution digital camera (1280×1024 pixels). All examinations were performed by the same trained technician using standardized operating procedures to minimize operational bias. The examination was conducted in a quiet, temperature- and humidity-controlled room (22-24 °C, 40%-60% relative humidity). All subjects rested in a sitting position with their hands at heart level for 20–30 minutes before the examination to eliminate the effects of temperature, exercise, and emotional stress on microcirculation. Subjects were instructed to avoid smoking, drinking caffeinated beverages, and engaging in strenuous physical activity for at least 2 hours before the examination. Raynaud’s phenomenon was excluded in all subjects through detailed medical history inquiry and physical examination of digital color changes in response to cold exposure. A drop of medical-grade cedarwood oil was applied to the nailfold skin of the index, middle, ring, and little fingers of both hands (thumbs were excluded due to high frequency of trauma and nail abnormalities). The distal capillary loops were observed at a magnification of 200×, and 3–5 representative images covering the entire nailfold area were captured for each finger. All images were independently interpreted by two rheumatologists who had received standardized training in nailfold capillaroscopy and had more than 3 years of clinical experience in this technique. Discrepancies between the two observers were resolved by consensus with a third senior rheumatologist with more than 10 years of experience. The inter-observer agreement was excellent, with a Cohen’s Kappa value of 0.86 (95% CI: 0.81-0.91). The NMC status was evaluated using the validated semi-quantitative scoring system proposed by Cutolo et al. (15), which includes three components with a total score ranging from 0 to 9 points: (a) Morphology score (0–3 points): 0 = all capillary loops are normal in shape and arrangement; 1 = <33% of loops show abnormalities (tortuosity, dilation, bushy loops, or megacapillaries); 2 = 33%-66% of loops are abnormal; 3 = >66% of loops are abnormal or avascular areas are present; (b) Flow pattern score (0–3 points): 0 = normal continuous blood flow; 1 = sluggish flow; 2 = intermittent flow; 3 = stasis or no visible blood flow; (C) Perivascular score (0–3 points): 0 = no perivascular changes; 1 = mild perivascular exudate; 2 = moderate exudate or single microhemorrhage; 3 = severe exudate or multiple hemorrhages. The total NMC score for each subject was calculated as the average score of the eight examined fingers.

The clinical data of the individuals were collected by reviewing electronic medical records, including gender, age, clinical manifestations, hematological indicators (mainly including complete blood count, erythrocyte sedimentation rate, C-reactive protein, etc.), immunological indicators (including peripheral blood Th1%, Th2%, Th17%, Treg%, etc.), and disease activity score (DAS28-ESR).

### Detection of Th17 and Treg cell subsets

Peripheral venous blood samples (2 mL) were collected in EDTA anticoagulant tubes and processed within 2 hours of collection to minimize cell viability loss. Peripheral blood mononuclear cells (PBMCs) were isolated by density gradient centrifugation using Ficoll-Hypaque solution (GE Healthcare, Chicago, IL, USA) at 400×g for 20 minutes at room temperature with the brake turned off. The interface layer containing PBMCs was collected and washed twice with phosphate-buffered saline (PBS) for subsequent staining. Instrumentation and quality control: Regulatory T (Treg) cell detection was performed on a Mindray BriCyto-E6 flow cytometer (Mindray Bio-Medical Electronics Co., Ltd., Shenzhen, China). Th17 cell detection was performed on a BD FACSCanto II flow cytometer (BD Biosciences, San Jose, CA, USA). All tests were performed by certified clinical laboratory technicians following strict standard operating procedures (SOPs). Instrument parameters (voltage, compensation) were calibrated daily using manufacturer-specific calibration beads. Fluorescence minus one (FMO) controls were used for all markers to set objective gating boundariesData analysis was performed using BriCyte Analysis Software (Mindray) for Treg cells and BD FACSDiva Software (BD Biosciences) for Th17 cells. Staining protocols and antibody panels: Treg cell panel: Freshly isolated PBMCs (1×10^6^ cells) were stained with the following surface antibodies for 30 minutes at 4 °C in the dark: CD3 (clone: UCHT1, PerCP-A), CD4 (clone: RPA-T4, FITC-A), CD25 (clone: M-A251, PE-A), and CD127 (clone: HIL-7R-M21, APC-A). No intracellular staining was required. Th17 cell panel: Freshly isolated PBMCs (1×10^6^ cells) were stained with the following surface antibodies for 30 minutes at 4 °C in the dark: CD4 (clone: RPA-T4, APC-Cy7-A), CD8 (clone: SK1, PerCP-Cy5.5-A), CD45RA (clone: HI100, FITC-A), CXCR3 (clone: 1C6/CXCR3, PE-A), and CCR6 (clone: 11A9, Pacific Blue-A). No intracellular staining or stimulation was required. Standardized gating strategy (representative plots shown in **Supplementary Figure 1**) (1): Treg cell gating: Lymphocyte gate was set based on forward scatter height (FSC-H) vs. side scatter height (SSC-H) dot plots to exclude non-lymphocyte events; CD3^+^ T cells were gated from the lymphocyte population; CD3^+^CD4^+^ helper T cells were further gated from the CD3^+^ T cell population; Treg cells were finally defined as CD3^+^CD4^+^CD25highCD127low/− cells, where CD25high was defined as the top 3% of CD25-expressing cells in the CD3^+^CD4^+^ T cell population (2). Th17 cell gating: Lymphocyte gate was set based on forward scatter area (FSC-A) vs. side scatter area (SSC-A) dot plots to exclude non-lymphocyte events; CD3^+^CD4^+^ helper T cells were gated from the lymphocyte population (CD8^+^T cells were excluded); CD45RA^−^ memory T cells were gated from the CD3^+^CD4^+^ T cell population to exclude naive T cells; Th17 cells were finally defined as CD3^+^CD4^+^CD45RA^−^CCR6^+^CXCR3^−^ cells. The percentage of both cell subsets was calculated relative to the total CD3^+^CD4^+^ T cells. The Th17/Treg ratio was calculated as the percentage of Th17 cells divided by the percentage of Treg cells.

### Statistical methods

Statistical analyses were performed using SPSS 27.0 software and R 4.4.1 software. Normally distributed quantitative data were expressed as mean ± SD, and comparisons between two groups were performed using the t-test. Non-normally distributed quantitative data were expressed as median (P25-P75), and comparisons between two groups were performed using the Mann-Whitney U test. Comparisons of enumeration data between the two groups were performed using the χ^2^ test. Spearman correlation analyses were used to assess the correlation between the total NMC score and PLR, PAR, SII, IL-2R, etc. To evaluate the diagnostic efficacy of the Th17/Treg ratio for moderate-to-severe NMC abnormalities, lasso regression and random forest models were constructed. Then a ROC curve was plotted, and the AUC and its 95% confidence interval (CI) were calculated. To evaluate the model’s performance, the Hosmer-Lemeshow test was used to assess the model’s goodness-of-fit (*p*>0.05 indicating a good degree of fitting). DCA was used to evaluate the clinical benefit of the model at different threshold probabilities. To test the stability of the model, the Bootstrap method was used for internal validation with 1000 repeated samplings to calculate the corrected average AUC value. *p* < 0.05 was considered statistically significant.

## Results

### Comparisons of NMC indicators between RA group and HC group

A total of 120 RA patients and 60 healthy controls were included in this study. The general demographic data of the two groups of subjects were compared and there was no statistical difference in age and gender composition between RA group and HC group (*p*>0.05) (**Supplementary Table 1**), demonstrating that the baseline data of the two groups were balanced.

**Figure 1:**
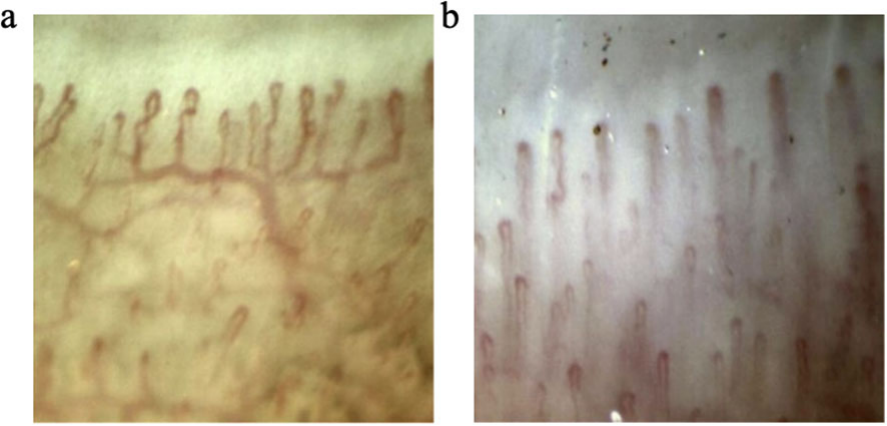
Representative NVC images for RA group **(a)** and HC group **(b)**.

Representative NVM images were presented in **Figure 1**. Based on the figures below, it is obvious that for an RA patient (**Figure 1a**), significant changes such as vessel crossing and abnormal angiogenesis could be observed compared to healthy controls (**Figure 1b**).

Regarding to NMV indicators, there existed significant differences between the RA group and the HC group. As shown in **Table 1**, the nailfold microcirculation of RA patients exhibited widespread and significant abnormalities. Compared to HC group, RA patients had a significantly increased number of crossed capillary loops, score for morphological abnormalities of loops, hemodynamic changes (incidence of slowed flow velocity and score for flow pattern abnormality), perivascular changes (perivascular score), and the total score reflecting the overall degree of lesions. All of these differences were statistically significant (*p* < 0.001), which indicated that RA patients generally suffer from obvious NMC disturbances.

**Table 1:** Comparisons of NMC indicators between RA group and HC group.

Indicators	RA group (n=120)	HC group (n=60)	Z/t value	*p*-value
Loop count	8.5 (7.0, 10.0)	9.0 (8.0, 10.0)	-0.103	0.918
Afferent limb diameter (µm)	5.0 (4.0, 7.0)	6.0 (6.0, 7.0)	-2.073	0.038
Efferent limb diameter (µm)	5.5 (4.0, 7.0)	6.0 (6.0, 7.0)	-2.681	0.007
Apical diameter (µm)	7.0 (6.0, 10.0)	8.0 (6.25, 10.00)	-1.394	0.163
Loop length (µm)	209.78 ± 72.2	203.10 ± 7.37	0.625	0.533
Crossed loop count	4.0 (2.0, 6.0)	2.0 (0, 3.0)	-5.153	<0.001
Morphology score	2.4 (1.63, 3.00)	1.6 (1.13, 1.80)	-5.43	<0.001
Flow velocity (µm/s)	500 (400, 500)	600 (525, 600)	-5.383	<0.001
Flow pattern score	1.2 (0.40, 2.85)	0 (0, 0.2)	-8.722	<0.001
Perivascular score	0.4 (0.1, 1.0)	0 (0, 0.4)	-5.844	<0.001
Total score	4.2 (3.4, 5.9)	1.8 (1.6, 2.0)	-9.056	<0.001

### Correlation analyses of NMC indicators with various inflammatory and immunological indicators in RA patients

**Figure 2:**
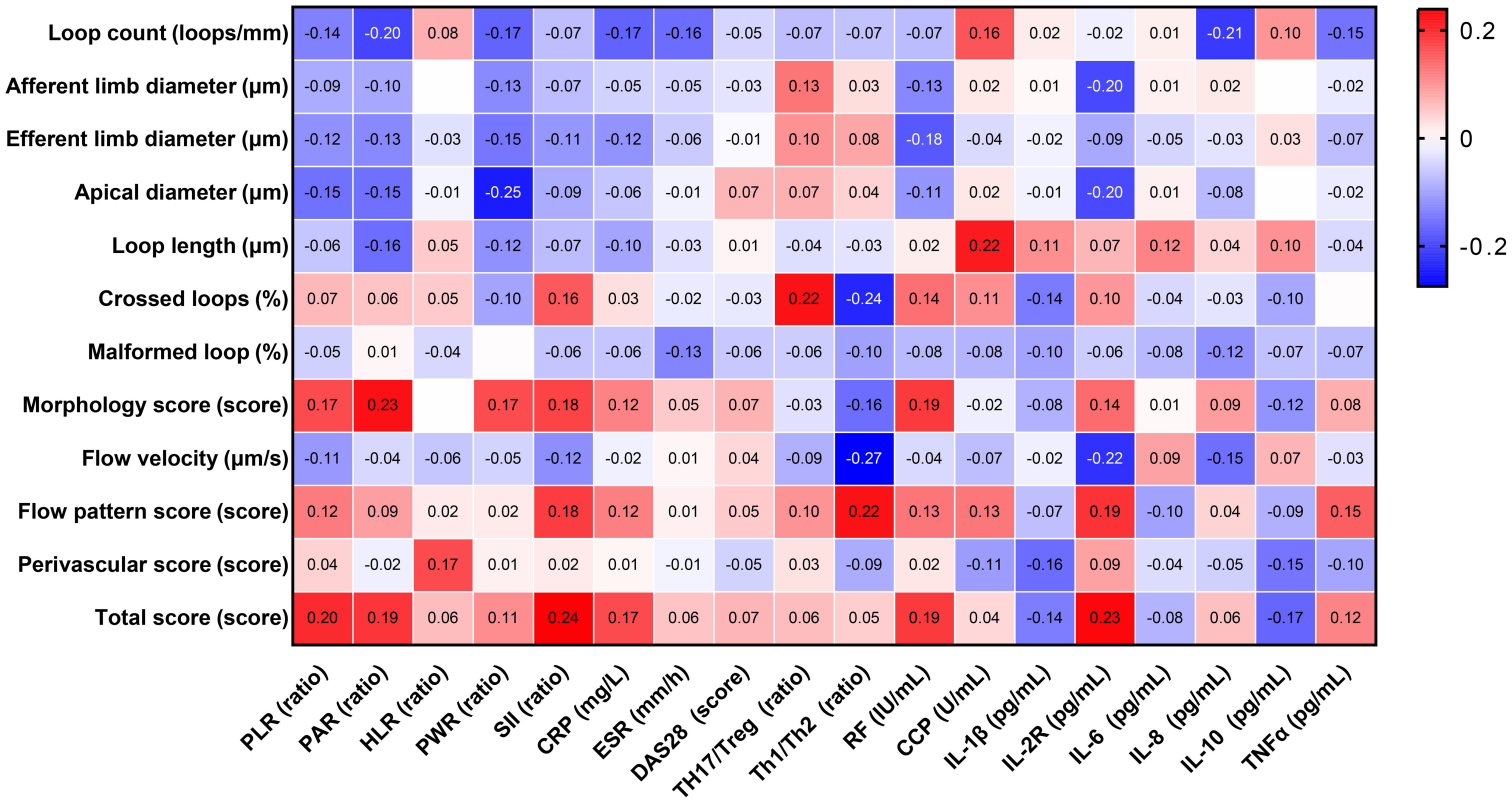
Correlation analyses of NMC indicators with various inflammatory and immunological indicators in RA patients.

To explore the potential factors driving microcirculatory damage in RA patients, we conducted Spearman correlation analyses between the NMC indicators of RA patients and a series of inflammatory or immunological indicators. The results, as shown in the **Figure 2**, indicated that NMC indicators showed significant correlations with multiple indicators reflecting systemic inflammatory burden and immune activation status. Among them, the correlation between the total NMC score and the SII was the strongest (*r* = 0.24, *p* = 0.009), suggesting that the SII, which integrates three cell lineages of neutrophils, lymphocytes, and platelets, could better reflect the systemic inflammatory state associated with microvascular damage. Secondly, the total NMC score also showed a significant positive correlation with soluble IL-2R (*r* = 0.23, *p* = 0.046), indicating that the degree of T-cell activation was directly related to the severity of microcirculatory disturbance. Furthermore, platelet-related inflammatory indicators, including PLR and PAR, were also significantly positively correlated with the total NMC score (*r* = 0.20, *p* = 0.028 and *r* = 0.19, *p* = 0.037, respectively), highlighting the important role of platelets in the vascular pathology of RA.

### Screening of indicators associated with moderate-to-severe NMC abnormalities

In this study, moderate-to-severe NMC abnormalities was defined as a total NMC score >4. This cut-off value was based on the internationally validated semi-quantitative scoring system for nailfold capillaroscopy proposed by Cutolo et al. (15), which classifies total scores of 0–2 as normal, 3–4 as mild abnormality, and >4 as moderate-to-severe abnormality. This classification has been widely adopted in clinical studies of rheumatic diseases (7, 16). In our cohort, the 95th percentile of the total NMC score in healthy controls was 2.0, and the median score in RA patients was 4.2, further supporting the rationality of this threshold. Sensitivity analysis showed that a cut-off value of 4.0 provided the best balance between sensitivity (62.1%) and specificity (72.2%) for identifying moderate-to-severe microvascular damage. Comparisons of hematological indices, systemic inflammation markers, acute-phase reactants, T-cell subset ratios, antibodies, and cytokines revealed a significant elevation of the Th17/Treg ratio in the moderate-to-severe NMC abnormalities group (**Table 2**). Subgroup analysis based on disease stage showed that there was no statistically significant difference in the total NMC score between newly diagnosed RA patients and long-standing RA patients (4.3 ± 1.8 vs. 4.1 ± 1.6, t=0.639, p=0.524). Similarly, the Th17/Treg ratio was also comparable between the two subgroups (6.12 ± 2.85 vs. 5.87 ± 2.61, t=0.507, p=0.613), indicating that the association between nailfold microcirculation abnormalities and Th17/Treg imbalance may not be affected by disease duration. Furthermore, one-way ANOVA analysis showed no significant differences in the total NMC score (*F=0.852, P = 0.472*) or Th17/Treg ratio (*F=0.0647, P = 0.589*) among the four treatment subgroups. *Post-hoc* pairwise comparisons also revealed no statistically significant differences between any two groups (all *p>0.05*). These results indicated that the current antirheumatic treatment regimens did not significantly confound the main association between nailfold microcirculation abnormalities and Th17/Treg imbalance observed in this study.

**Table 2:** Comparisons of different indices between mild NMC abnormalities and moderate-to-severe NMC abnormalities.

Variable	Mild NMC abnormalities (n=54)	Moderate-to-severe NMC abnormalities (n=66)	Z/t value	*p*-value
Hematological indices				
WBC (10^9^/L)	5.88 (4.27-7.40)	5.98 (4.69-8.13)	1.295	0.196
RBC (10^12^/L)	4.17 (3.80-4.48)	4.02 (3.75-4.33)	1.000	0.319
HGB (g/L)	124.50 (112.25-132.75)	121.50 (113.00-133.75)	0.208	0.837
PLT (10^9^/L)	228.50 (206.00-277.25)	243.00 (190.50-323.00)	0.593	0.555
Neutrophils (10^9^/L)	3.45 (2.35-4.83)	3.76 (2.63-5.31)	1.073	0.284
Lymphocytes (10^9^/L)	1.57 (1.16-1.97)	1.52 (1.14-1.93)	0.076	0.941
Systemic inflammation				
NLR	2.19 (1.72-3.09)	2.43 (1.53-4.12)	0.739	0.462
PLR	154.95 (122.92-203.90)	153.39 (121.74-219.21)	0.422	0.675
PAR	56.56 (48.32-70.66)	60.26 (46.62-79.87)	0.739	0.462
HLR	76.75 (63.27-102.11)	80.76 (60.00-113.39)	0.372	0.712
PWR	37.24 (32.70-54.25)	40.94 (31.89-51.93)	0.095	0.926
SII	574.51 (356.28-765.32)	572.04 (341.31-1065.45)	0.633	0.528
Acute-phase reactants and disease activity				
CRP (mg/L)	6.17 (3.64-22.10)	8.57 (3.64-29.10)	0.477	0.634
ESR (mm/h)	28.50 (15.00-49.50)	27.00 (14.00-50.00)	0.182	0.858
DAS28 score	4.48 ± 1.36	4.77 ± 1.15	1.249	0.214
Ratio of T cell subsets				
Th17/Treg ratio	4.92 (4.11-5.71)	6.95 (4.96-9.49)	4.676	<0.001***
Th1/Th2 ratio	1.24 (0.92-1.88)	1.37 (1.05-1.59)	0.570	0.571
Antibodies				
RF (IU/mL)	59.90 (17.60-211.00)	64.95 (18.38-235.25)	0.108	0.916
Anti-CCP antibody (U/mL)	100.02 (29.25-200.00)	123.40 (32.01-200.00)	0.488	0.623
Cytokines				
IL-1β (pg/mL)	5.00 (5.00-8.43)	5.00 (5.00-7.26)	1.279	0.137
IL-2R (pg/mL)	512.50 (423.50-726.75)	572.00 (386.50-800.75)	0.746	0.457
IL-6 (pg/mL)	12.20 (3.40-50.70)	10.65 (3.03-29.28)	1.255	0.21
IL-8 (pg/mL)	85.05 (19.78-484.00)	38.10 (15.70-228.00)	1.754	0.08
IL-10 (pg/mL)	5.00 (5.00-5.00)	5.00 (5.00-5.00)	1.097	0.057
TNF-α (pg/mL)	25.15 (15.38-150.00)	20.35 (14.65-143.25)	0.841	0.401

**Figure 3:**
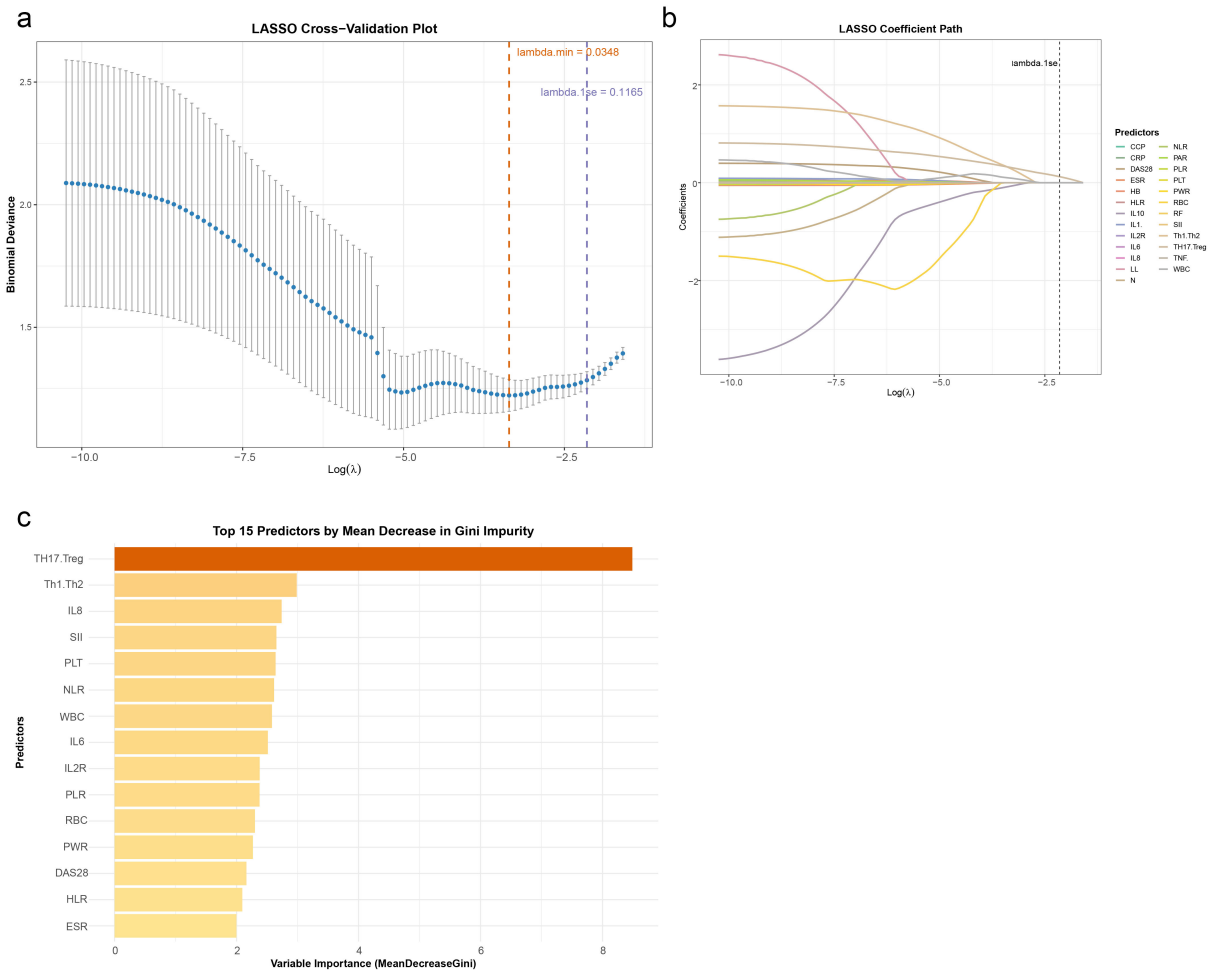
LASSO regression analysis and random forest model to identified the indicators of moderate-to-severe NMC abnormalities: **(a)** LASSO cross-validation plot; **(b)** LASSO coefficient path; **(c)** feature importance ranking based on a random forest model.

To identify key predictors, these variables were subjected to LASSO regression analysis. Upon selection of the optimal λ value (**Figure 3a**), the Th17/Treg ratio was identified as the primary predictive variable (**Figure 3b**). Furthermore, feature importance ranking based on a random forest model confirmed that the Th17/Treg ratio was the main factor influencing moderate-to-severe NMC abnormalities (**Figure 3c**).

### Analyses of the diagnostic value of the Th17/Treg ratio for moderate-to-severe NMC abnormalities

**Figure 4:**
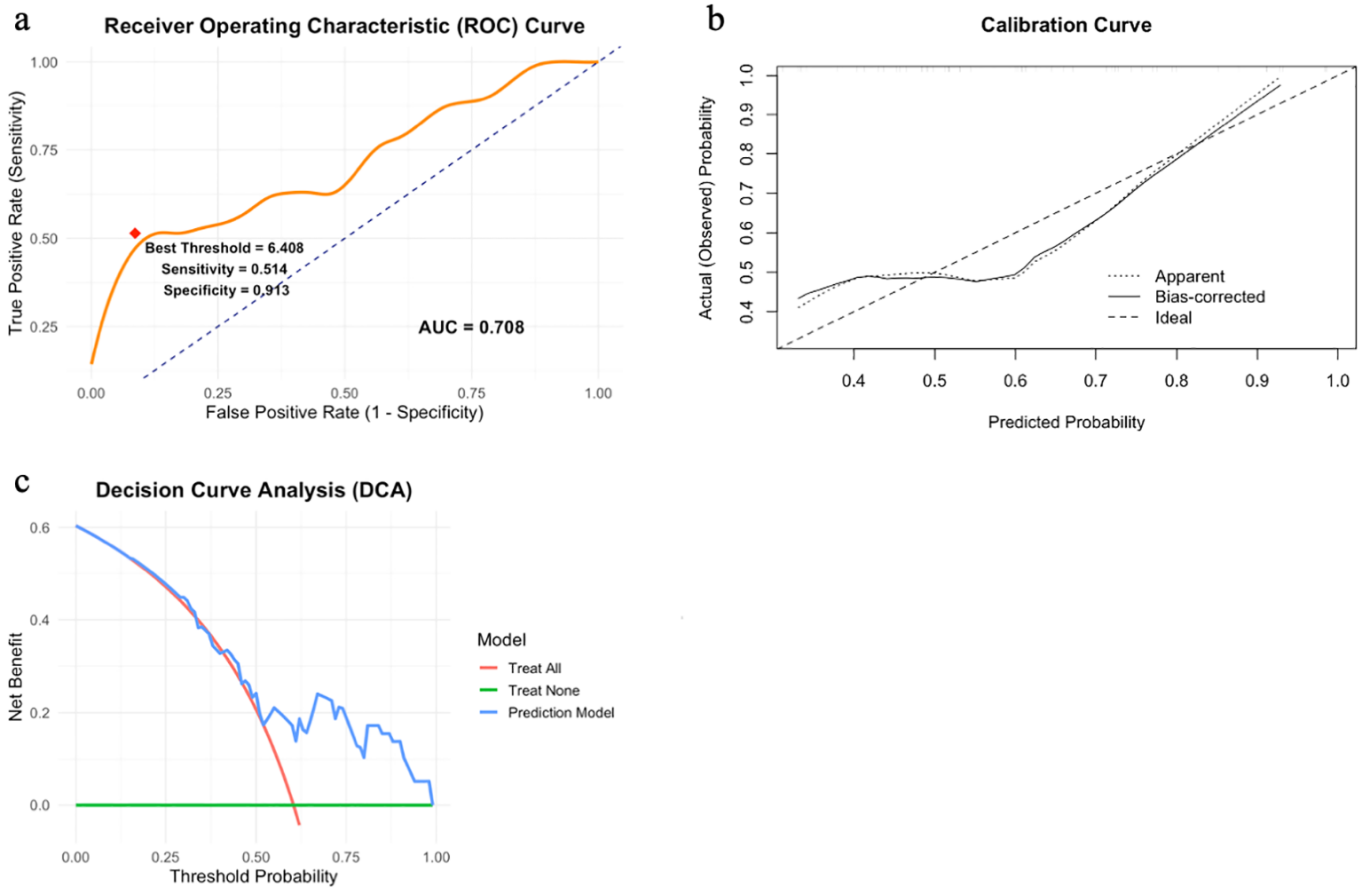
Analyses of the diagnostic value of the Th17/Treg ratio for moderate-to-severe NMC abnormalities: **(a)** ROC curve for Th17/Treg ratio in diagnosing moderate-to-severe NMC abnormalities; **(b)** calibration curve for above model; **(c)** DCA curve for above model.

Given the core position of Th17/Treg immune imbalance in the pathogenesis of RA, this study further explored the diagnostic value of this ratio in predicting severe microcirculatory lesions. We defined a total NMC score greater than 4 as moderate-to-severe microcirculatory abnormality and constructed a logistic regression model based on the Th17/Treg ratio. The results showed that AUC for this model was 0.708 (95% CI: 0.615-0.801, *p* < 0.001), suggesting that this indicator had moderate-to-high diagnostic accuracy (**Figure 4a**).

To comprehensively evaluate the performance of this model, we conducted a Hosmer-Lemeshow test and the *p*-value for the goodness-of-fit test was 0.657 (>0.05), indicating that the model had a considerable fitness. The calibration curve also visually demonstrated that the curve of the model’s predicting probabilities versus the actual occurrence probabilities was generally distributed along the diagonal line, further confirming the calibration ability of the model (**Figure 4b**). The results of the DCA showed that the model possessed high-level clinical practicality (**Figure 4c**). Finally, to assess the robustness of the model, we used the Bootstrap method to perform internal validation with 1000 repeated samplings. The validation results showed that the corrected average AUC was 0.706, which was very close to the original AUC value, indicating that the model’s predictive performance was stable and reliable, and not easily affected by sampling variation.

## Discussions

The results of this study showed that, compared to the HC group, the NMC of RA patients had severe abnormalities in both structure and function, which is consistent with previous reports (16). The changes observed under NVC, such as twisted capillary loops, slowed blood flow, and perivascular exudate, are direct morphological evidences of vascular endothelial cell injury, increased permeability, and dysfunction. They are also initiating factors for the development of macrovascular atherosclerosis. Previous studies have confirmed that changes in skin microcirculation are associated with macroscopic circulatory indicators such as the perfusion status of the coronary microcirculation and the stiffness of large arteries. Therefore, the severe NMC abnormalities quantitatively assessed by NVC in this study are highly likely not just a local phenomenon, but rather an indicator of the systemic vascular disease.

This study quantitatively correlated observable microcirculatory damage with the intrinsic immune-inflammatory states that drive this damage. The positive correlation between the total NMC score and hematological indicators such as SII, PLR, and PAR suggests a potential direct link between systemic inflammatory burden and peripheral vascular damage. High levels of SII and PLR imply more activated platelets and neutrophils in circulation. Activated platelets not only participate in thrombosis through aggregation but, more importantly, act like “mobile cytokine depots”, releasing large quantities of pro-inflammatory mediators and growth factors (17). Neutrophils, on the other hand, directly inflict oxidative stress and proteolytic damage on vascular endothelial cells by releasing reactive oxygen species (ROS) and proteolytic enzymes. The interaction of these cells with the vascular endothelium induces high expression of adhesion molecules on endothelial cells, thereby recruiting more inflammatory cells to infiltrate the vessel wall, forming a vicious cycle known as “type II endothelial cell activation” (18). This long-term, chronic inflammatory assault may ultimately lead to structural remodeling of the vessel wall, which manifests as abnormalities in loop morphology under NVC, increased vascular permeability (perivascular exudate, hemorrhage), and the appearance of pathological, disordered angiogenesis for damage repair.

The positive correlation between the total NMC score and IL-2R, in turn, directly linked T-cell activation, a core event of adaptive immunity, with vascular damage. IL-2R is one of the “gold standards” for T-cell activation, and its level directly reflects the intensity of the T-cell-mediated immune response (19). The initiation and development of RA are rooted in the abnormal reaction and persistent activation of T cells against self-antigens. Activated T cells not only shed IL-2R into the circulation themselves but, more importantly, as the “commanders” of the immune system, they mobilize and direct the entire immune system’s attack behavior by secreting a series of cytokines such as interferon-γ (IFN-γ) and tumor necrosis factor-α (TNF-α). These cytokines are potent mediators that induce endothelial dysfunction. They can act directly on vascular endothelial cells, causing them to transition from a normal resting state to a pro-inflammatory, pro-coagulant, and hyperpermeable pathological state (20). Therefore, a higher serum IL-2R level indicates stronger T-cell activation, which may be associated with more intense vascular inflammatory responses and ultimately manifest as more severe microvascular damage and a higher NMC score.

One of the most innovative findings of this study is the confirmation that the Th17/Treg ratio could effectively predict moderate-to-severe NMC abnormalities (AUC = 0.708). This result revealed a profound association between specific subtypes of immune imbalance and specific pathological damage. The dysregulation of the Th17/Treg balance represents a unique pro-inflammatory pathway centered on the IL-17 cytokine family (21). A key and unique biological effect of IL-17 is its potent promotion of angiogenesis and efficient recruitment of neutrophils to inflammatory sites (22). Under NVC observation, pathological angiogenesis manifests as disorganized, bizarrely shaped, bushy or antler-like capillary loops, which are an important component constituting severe NMC abnormalities and high scores. Meanwhile, the functionally deficient or numerically insufficient Treg cells in RA patients cannot effectively suppress Th17 cell-driven pathological inflammation, leading to persistent and worsening vascular damage (23). Therefore, a high Th17/Treg ratio is associated with an immune phenotype that may preferentially contribute to vascular structural damage and pathological angiogenesis, which explains why this ratio can relatively specifically predict moderate-to-severe NMC abnormalities. It suggests to us that for those RA patients who simultaneously exhibit a high Th17/Treg ratio and severe NMC abnormalities, the driving mechanism of their vascular lesions may be dominated by the IL-17 pathway.

This study also has some limitations. Firstly, as a single-center, cross-sectional observational study, its results mainly reveal statistical associations between variables and cannot yet determine the causal relationships, temporal sequence, nor the direction of the association between nailfold microcirculation abnormalities and systemic inflammation or Th17/Treg dysregulation. All findings in this study are strictly limited to statistical associations. No causal inferences can be drawn regarding whether systemic inflammation or Th17/Treg dysregulation causes nailfold microcirculation abnormalities, or vice versa. In the future, we will conduct a 3-year prospective follow-up study to observe the dynamic evolution of nailfold microcirculation indicators and related biomarkers in RA patients, and further verify their independent predictive value for future cardiovascular events and disease progression. Prospective interventional studies are also required to determine whether modulating these inflammatory or immunological factors can improve nailfold microcirculation and reduce cardiovascular risk in RA patients. Secondly, all subjects enrolled in this study were Han ethnicity from northern China, and the single-center design may lead to selection bias, limiting the generalizability of the results to other ethnic groups and regions. A multi-center, multi-ethnic collaborative study with a larger sample size is needed to validate and extend our findings in subsequent studies. Thirdly, although the sample size of this study was calculated based on pre-experimental data and the statistical power of the main conclusions was sufficient, a larger cohort is still required to further confirm the stability of the Th17/Treg ratio prediction model and explore the potential differences in nailfold microcirculation characteristics among different RA subgroups (e.g., patients with different autoantibody statuses or extra-articular manifestations). Fourthly, most of the enrolled patients had moderate disease activity (54.2% with DAS28-ESR 3.2-5.1), and only 22.5% were in remission or low activity. No patients receiving long-term deep remission treatment (DAS28<2.6 for ≥6 months) were included in this study. This may explain why no significant differences in nailfold microcirculation severity or Th17/Treg ratio were found among different treatment subgroups: the underlying immune imbalance and vascular endothelial damage had not been fully reversed by the current short-term or suboptimal therapies. Future studies should focus on the dynamic changes of nailfold microcirculation after targeted therapy, especially the effect of IL-17/IL-23 axis inhibitors on improving microvascular lesions, which may provide a new basis for personalized vascular protection strategies in RA patients.

Despite these limitations, our finding has significant potential value for clinical translation. We revealed that the severity of NMC status in patients with RA was significantly associated with systemic inflammatory burden and T-cell immune activation, and this association was consistent across different disease stages and treatment subgroups. This suggests that nailfold microcirculation assessment may be a valuable tool for evaluating vascular risk in RA patients regardless of disease duration or treatment status. In terms of surveillance and treatment selections, our study suggests that monitoring IL-2R levels might help identify RA subpopulations that are at high risk for vascular damage due to high T-cell activation. What’s more, biologic agents targeting the IL-17/IL-23 axis, or innovative therapies aimed at restoring Treg cell function, may offer more targeted and effective vasoprotective effects for RA patients with high Th17/Treg ratios and severe NMC abnormalities. Future prospective interventional studies are warranted to validate whether these targeted strategies can indeed improve microcirculation and reduce cardiovascular events in this high-risk population.

## Conclusions

This study systematically evaluated the NMC status of patients with RA and innovatively correlated it with a series of novel systemic inflammatory and immunological indicators, as well as the imbalance status of core T-cell subpopulations. The study illustrated that RA patients universally had significant NMC disturbances and the severity of these disturbances was significantly associated with indicators reflecting systemic inflammatory burden (SII, PLR, PAR) and T-cell immune activation (IL-2R, Th17/Treg ratio). These findings not only provide new evidence for a deeper understanding of the vascular pathophysiology of RA but also suggest potential new avenues for assessing vascular lesions and conducting risk stratification in clinical practice.

## Data Availability

The original contributions presented in the study are included in the article/**Supplementary Material**. Further inquiries can be directed to the corresponding authors.

## Supplementary material

Supplementary Figure 1 Representative flow cytometry gating strategy for Th17 and Treg cell subsets. **(A)** Gating strategy for Treg cells (detected on Mindray BriCyto-E6 flow cytometer) **(a)** Lymphocyte gate (P1) was set based on FSC-H vs. SSC-H dot plots to exclude cell debris and non-lymphocyte events. **(b)** CD3^+^ T cells were gated from the lymphocyte population. **(c)** CD3^+^CD4^+^ helper T cells (P2) were further gated from the CD3^+^ T cell population. **(d)** Treg cells were identified as CD25highCD127low/− cells (P3) within the CD3^+^CD4^+^ T cell gate. The percentage of Treg cells was 8.08% relative to CD3^+^CD4^+^ T cells. **(B)** Gating strategy for Th17 cells (detected on BD FACSCanto II flow cytometer) **(a)** Lymphocyte gate was set based on FSC-A vs. SSC-A dot plots to exclude non-lymphocyte events. **(b)** CD3^+^CD4^+^ helper T cells (purple population) were gated from the lymphocyte population, and CD8^+^ T cells (green population) were excluded. **(c)** CD45RA^−^ memory T cells were gated from the CD3^+^CD4^+^ T cell population to exclude naive T cells. **(d)** Th17 cells were identified as CCR6^+^CXCR3^−^ cells (lower right quadrant) within the CD3^+^CD4^+^CD45RA^−^ memory T cell gate. All gating boundaries were determined using fluorescence minus one (FMO) controls. The numbers in each gate indicate the percentage of the corresponding cell population relative to its parent population.
