# Supplementary figures and images for: The associations between nailfold microvascular abnormalities and systemic inflammation or Th17/Treg dysregulation in rheumatoid arthritis
Source: Front Immunol. 2026 Jun 26;17:1848275. doi: 10.3389/fimmu.2026.1848275 (PMC13349768; doi:10.3389/fimmu.2026.1848275)

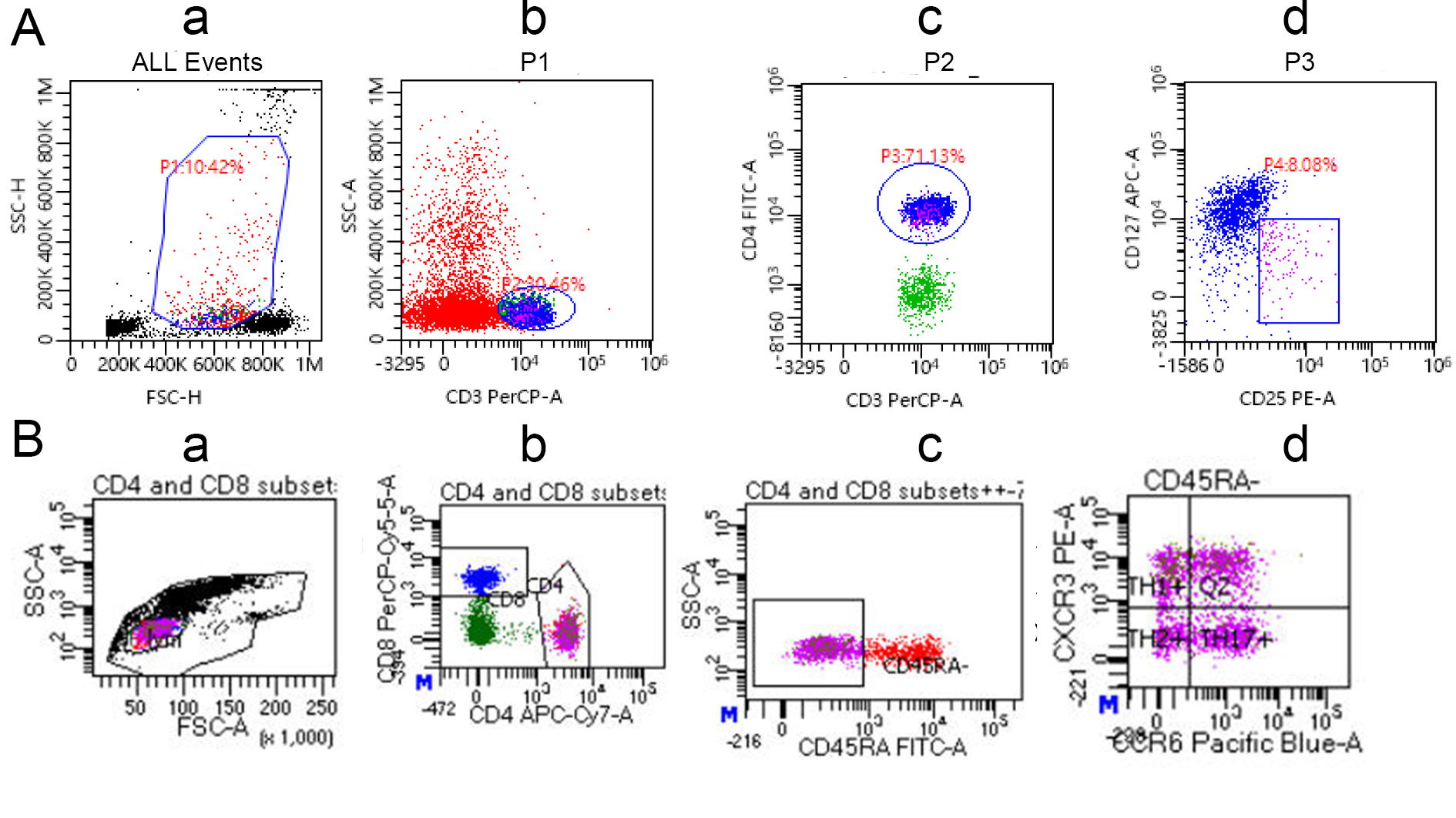

Supplement: Supplementary Figure 1 — Representative flow cytometry gating strategy for Th17 and Treg cell subsets. (A) Gating strategy for Treg cells (detected on Mindray BriCyto-E6 flow cytometer) (a) Lymphocyte gate (P1) was set based on FSC-H vs. SSC-H dot plots to exclude cell debris and non-lymphocyte events. (b) CD3+ T cells were gated from the lymphocyte population. (c) CD3+CD4+ helper T cells (P2) were further gated from the CD3+ T cell population. (d) Treg cells were identified as CD25highCD127low/− cells (P3) within the CD3+CD4+ T cell gate. The percentage of Treg cells was 8.08% relative to CD3+CD4+ T cells. (B) Gating strategy for Th17 cells (detected on BD FACSCanto II flow cytometer) (a) Lymphocyte gate was set based on FSC-A vs. SSC-A dot plots to exclude non-lymphocyte events. (b) CD3+CD4+ helper T cells (purple population) were gated from the lymphocyte population, and CD8+ T cells (green population) were excluded. (c) CD45RA− memory T cells were gated from the CD3+CD4+ T cell population to exclude naive T cells. (d) Th17 cells were identified as CCR6+CXCR3− cells (lower right quadrant) within the CD3+CD4+CD45RA− memory T cell gate. All gating boundaries were determined using fluorescence minus one (FMO) controls. The numbers in each gate indicate the percentage of the corresponding cell population relative to its parent population. [file Image1.jpeg]
