# Supplementary material for: The associations between nailfold microvascular abnormalities and systemic inflammation or Th17/Treg dysregulation in rheumatoid arthritis
Source: Front Immunol. 2026 Jun 26;17:1848275. doi: 10.3389/fimmu.2026.1848275 (PMC13349768; doi:10.3389/fimmu.2026.1848275)
Supplement: Supplementary file 2 [file Table1.docx]

**Supplementary Table 1: Comparison of general characteristics between RA group and HC group**

| **Characteristics** | **RA group (n=120)** | **HC group (n=60)** | **Statistical value** | **pvalue** |
| --- | --- | --- | --- | --- |
| Demographic characteristics |  |  |  |  |
| Age (years), mean±SD | 62.4 ± 10.6 | 60.0 ± 7.9 | t=1.805 | 0.073 |
| Gender (male/female), n | 24/96 | 18/42 | χ²=2.174 | 0.140 |
| Disease characteristics |  |  |  |  |
| Disease course (months), median (IQR) | 36.0 (12.0, 84.0) |  |  |  |
| DAS28ESR score, mean±SD | 4.64±1.26 |  |  |  |
| Disease activity (DAS28ESR), n (%) |  |  |  |  |
| Remission/low activity (<3.2) | 27 (22.5) |  |  |  |
| Moderate activity (3.25.1) | 65 (54.2) |  |  |  |
| High activity (≥5.1) | 28 (23.3) |  |  |  |
| Disease stage, n (%) |  |  |  |  |
| Newly diagnosed RA | 38 (31.7) |  |  |  |
| Longstanding RA | 82 (68.3) |  |  |  |
| Treatment status, n (%) |  |  |  |  |
| Untreated | 38 (31.7) |  |  |  |
| csDMARDs monotherapy | 42 (35.0) |  |  |  |
| Methotrexate (MTX) | 28 (66.7) |  |  |  |
| Leflunomide (LEF) | 10 (23.8) |  |  |  |
| Sulfasalazine (SASP) | 4 (9.5) |  |  |  |
| csDMARDs + lowdose glucocorticoids | 26 (21.7) |  |  |  |
| Prednisone ≤10 mg/day (equivalent dose) | 26 (100.0) |  |  |  |
| b/tsDMARDsbased therapy | 14 (11.7) |  |  |  |
| TNF inhibitors (TNFi) | 8 (57.1) |  |  |  |
| IL6 inhibitors (IL6i) | 4 (28.6) |  |  |  |
| JAK inhibitors (JAKi) | 2 (14.3) |  |  |  |
| Extraarticular manifestations, n (%) | 18 (15.0) |  |  |  |
| Interstitial lung disease | 7 (5.8) |  |  |  |
| Rheumatoid nodules | 6 (5.0) |  |  |  |
| Ocular involvement | 3 (2.5) |  |  |  |
| Others | 2 (1.7) |  |  |  |
